# An uncommon source of upper gastrointestinal bleeding: epiphrenic esophageal diverticulum

**DOI:** 10.1093/gastro/gov043

**Published:** 2015-08-20

**Authors:** Chung Sang Tse, Neil D Parikh

**Affiliations:** 1 Department of Internal Medicine, Mayo Clinic, Rochester, MN, USA; 2 Division of Gastroenterology, Department of Internal Medicine, Yale School of Medicine, New Haven, CT, USA

**Keywords:** upper gastrointestinal bleed, epiphrenic esophageal diverticulum, esophago-gastroduodenoscopy

## Abstract

Esophageal diverticula are rare findings that have an estimated incidence of 1 per 500 000 people per year, even though acute upper gastrointestinal bleeding is a relatively common medical emergency with an incidence of up to 150 per 100 000 people per year and a mortality rate of 7–14%. An 83-year-old man presented with hematemesis and melena. Urgent upper endoscopy revealed an esophageal diverticulum, within which was an adherent clot. Removal of the clot identified a bleeding vessel within the diverticulum; this was successfully clipped and hemostasis was achieved. To the best of our knowledge, this is the first case report of a bleeding epiphrenic esophageal diverticulum that was successfully managed endoscopically with hemostatic clips alone. While rare, our case serves as a reminder that bleeding epiphrenic esophageal diverticula can present as massive upper gastrointestinal bleeding and urgent endoscopic therapy can be life-saving.

## Case presentation

An 83-year old man presented to our emergency department with lightheadedness, multiple episodes of melena, and hematemesis. He had a medical history of hypertension, abdominal aortic aneurysm repair, daily use of naproxen for arthritic joint pain, and heavy alcohol consumption. On arrival, his blood pressure was 118/67 mmHg, heart rate 97 beats per minute, and he was positive for orthostatic hypotension. Laboratory tests revealed a hemoglobin level of 11.4 g/dL (normal range 14.0–18.0 g/dL), hematocrit of 32.7% (normally 40.0–52.0%), blood urea nitrogen of 32 mg/dL (normally 7–20 mg/dL), creatinine 1.3 ml/dL (normally 0.5–1.2 ml/dL), and blood urea nitrogen to creatinine ratio of 24.6 (normal range 10.0–20.0). His initial Rockall Score was 2 (out of 7). Initial management in the Emergency Department included volume resuscitation with normal saline boluses and continuous infusion of pantoprazole 8.0 mg/h. The patient was admitted to the step-down unit. Subsequently, he had three additional episodes of hematemesis. Hemoglobin deteriorated to 6.6 g/dL with a hematocrit of 18.9%. His blood pressure reached a nadir of 60/30 mmHg. He was started on a peripheral dopamine infusion for blood pressure support and received a transfusion of three units of packed red blood cells. At this point, in light of the patient’s medical history, the suspected sources of the upper gastrointestinal bleeding included peptic ulceration, mucosal tear, ruptured esophageal varix, and arterial-venous malformation, among others.

**Figure 1.**
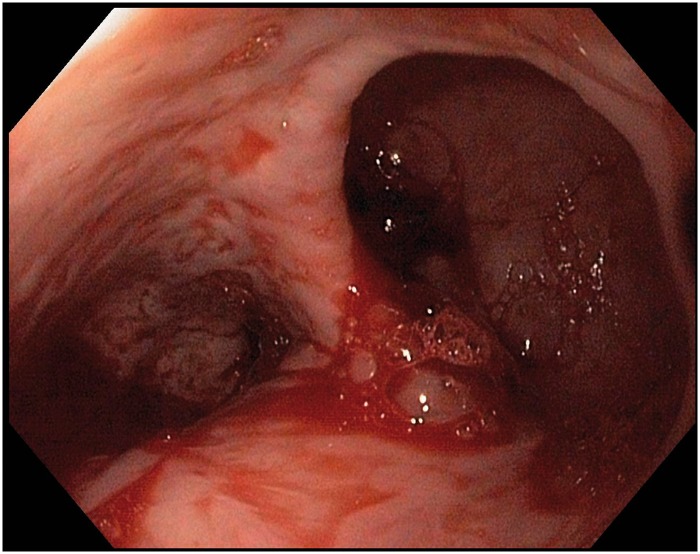
Bleeding epiphrenic esophageal diverticulum. At the distal portion of the esophagus, a wide-mouth diverticulum (right) with an oozing blood vessel was seen via esophago-gastroduodenoscopy.

**Figure 2.**
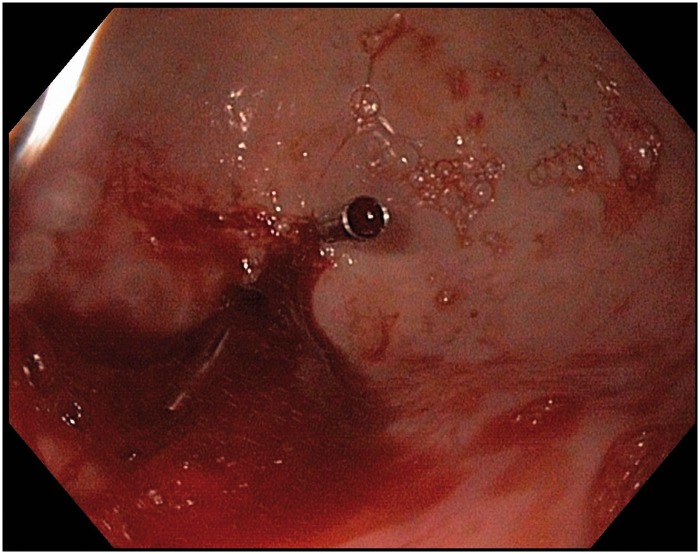
Bleeding epiphrenic diverticulum after endoscopic clipping. Two hemostatic clips were applied to the oozing vessel within the epiphrenic diverticulum. Hemostasis was achieved after the second clip (center) was placed. A residual blood clot is observed in the lower left corner.

Urgent upper endoscopy was performed nine hours after admission, which revealed a broad-mouthed, right-sided diverticulum in the distal esophagus. At the base of the diverticulum, an adherent blood clot was visualized and it was washed off by targeted jet irrigation, revealing an actively oozing blood vessel (Figure 1). Two hemostatic clips (Resolution™ Clip, ref. M00522612; Boston Scientific, Boston MA, USA) were placed directly on the bleeding vessel and hemostasis was achieved (Figure 2). The patient's post-procedure Rockall Score was 7 (out of 11). Overnight, the patient had three additional episodes of melena but no hematemesis. The following day he was hemodynamically stable, off vasopressors, and resumed an oral diet. His hemoglobin was 8.5 g/dL and hematocrit was 24%, both of which remained stable for the remainder of his hospitalization. He was discharged home two days after the endoscopy procedure and remained asymptomatic one month after. Outpatient follow-up appointments were established at our institution’s gastroenterology clinic; however, the patient cancelled the appointments and was only reachable by phone. A barium esophagram was ordered to further evaluate the diverticulum and the hemostasis clips, but the patient declined as he was feeling well.

## Discussion

Esophageal diverticula are rare findings that have an estimated incidence of 1:500 000 per year and a prevalence of 0.015–2% [1–4]. Most esophageal diverticula are acquired lesions that present in older adults [5], the average patient age of diagnosis is 60–70 years old [6], and there is a male predominance with a male–female ratio of around 2–3:1 [7]. Epiphrenic esophageal diverticula are located 10 cm from the gastroesophageal junction and account for about 15% of all esophageal diverticulae; the majority are false diverticula, containing the mucosal and submucosal layers without the *muscularis propria* [6]. Epiphrenic esophageal diverticula more commonly occur on the right side, perhaps because the aorta and the heart inhibit the enlargement of diverticula on the left side [7, 8]. The mechanism of formation of epiphrenic esophageal diverticula is most commonly attributed to pulsion forces associated with esophageal dysmotility, functional or mechanical obstruction, and local wall weakness leading to mucosal herniation at the segment of maximal luminal pressure and least wall resistance [6]. Symptoms of epiphrenic esophageal diverticula are most commonly intermittent dysphagia and regurgitation although, as with our patient, up to 80% of patients have minimal symptoms or none at all [5, 6, 9]. Malignancy, particularly squamous cell carcinoma, is a rare complication of esophageal diverticula [5].

To the best of our knowledge, this is the first case report of a patient with a bleeding epiphrenic diverticulum, who was managed endoscopically with hemostatic clips alone. Three previous case reports of successful endoscopic management of this condition involved epinephrine injection with thermal coagulation [5], epinephrine injection with hemostatic clip [10], or thermal coagulation alone [11]. We chose placement of a hemostatic clip over thermal coagulation, given the clearly visible bleeding vessel. In general, we prefer clip placement over thermal coagulation because of its fewer post-procedural complications, such as ulcer formation; also we did not use epinephrine injection alone as a single hemostasis modality due to its significantly greater re-bleeding rates as compared with combined therapy with clip placement or coagulation [12]. Surgical management was historically the mainstay of treating epiphrenic diverticulum prior to the widespread adoption of interventional endoscopy [7]. Surgical procedures include laparoscopic diverticulectomy, myomectomy, or anti-reflux procedure (such as Nissen-Rossetti fundoplication) [8]. Surgical evaluation was not necessary in our patient as effective hemostasis was achieved endoscopically. Our case supports previous literature suggesting that endoscopic treatment should be considered as first-line treatment of bleeding epiphrenic esophageal diverticula [5].

*Conflict of interest statement*: none declared.
